# Farming Behavior by the Large Hawk‐Cuckoo Toward the Silver‐Eared Mesia and Black‐Streaked Scimitar Babbler

**DOI:** 10.1002/ece3.72955

**Published:** 2026-01-09

**Authors:** Feiling Pang, Keyan Zhang, Ziyu Yuan, Jianping Liu

**Affiliations:** ^1^ College of Biological Sciences and Engineering North Minzu University Yinchuan China

**Keywords:** black‐streaked scimitar babbler, brood parasitism, farming behavior, large hawk‐cuckoo, silver‐eared Mesia

## Abstract

Brood parasites improve their reproductive success by offloading the costs of incubation and chick‐rearing onto host species. This interaction often triggers an evolutionary arms race between increasingly deceptive brood parasites and increasingly defensive hosts. According to the farming hypothesis, when suitable host nests are limited, some parasitic birds may destroy unsuitable host nests through “Farming behavior” to induce re‐nesting, thereby creating new opportunities for parasitism. Despite its potential significance, this behavior has been documented in only a few brood‐parasitic species. In 2024, in Jindong County, Pu'er City, Yunnan Province, southwestern China, we successfully captured video evidence of Farming behavior by the Large Hawk‐cuckoo (*Hierococcyx sparverioides*) on its host, the Black‐streaked Scimitar Babbler ( *Pomatorhinus erythrocnemis* ), and potential host, the Silver‐eared Mesia ( *Leiothrix argentauris* ). On April 22, 2024, a Large Hawk‐cuckoo was recorded removing a 7‐day‐old Black‐streaked Scimitar Babbler chick from its nest, but the chick subsequently crawled back inside. On May 27, 2024, a Large Hawk‐cuckoo was observed ejecting three 10‐day‐old Silver‐eared Mesia chicks from their nest. This is the first recorded instance of the Large Hawk‐cuckoo removing chicks from the nests of both the host and potential host species. The observed actions are consistent with the Farming hypothesis, suggesting that such behavior may be a more widespread yet underrecognized strategy among brood‐parasitic birds.

## Graphical abstract

Some parasitic birds may destroy unsuitable host nests through “Farming behavior” to induce re‐nesting, thereby creating new opportunities for parasitism. Despite its potential significance, this behavior has been documented in only a few brood‐parasitic species. This is the first recorded instance of the Large Hawk‐cuckoo removing chicks from the nests of both the host (Black‐streaked Scimitar Babbler *Pomatorhinus erythrocnemis* ) and potential host (Silver‐eared Mesia *Leiothrix* *argentauris*).

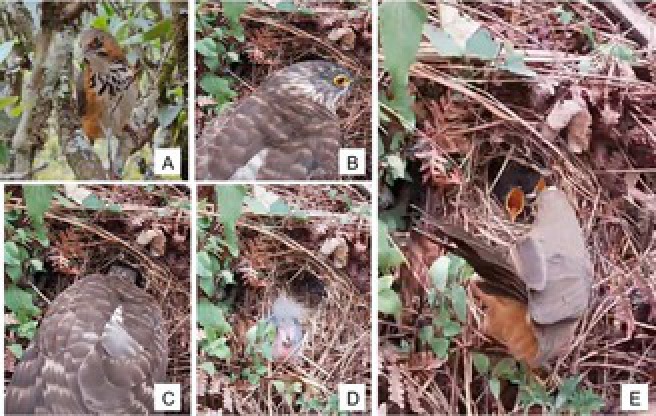

## 1 Introduction

Interactions between species are a major driver of evolution and biological diversity. In many cases, antagonistic interactions involve ongoing cycles of adaptation and counter‐adaptation between different species, resulting in coevolution. Coevolutionary interactions are widespread in nature (Thompson 2005). One fascinating example of this is the phenomenon of brood parasitism, wherein brood parasites do not build nests themselves and lay their eggs in host nests. The host subsequently raises the unrelated chicks, often at the cost of its own offspring's survival (Davies 2000; Soler 2014).

Brood parasites exert strong selection pressure on hosts, forcing them to evolve a range of antiparasitic adaptations, such as the ability of some hosts to recognize and reject parasitized eggs and chicks (Langmore et al. 2003; Attisano et al. 2021; Liu et al. 2023; Lund et al. 2023; Noh et al. 2023; Yan et al. 2025). Certain hosts reduce the risk of parasitism by synchronizing their breeding to reduce the chance of being targeted (Payne 1973; Martinez et al. 1996; Massoni and Reboreda 2001; Jelínek et al. 2014). However, when brood parasites miss the peak of host egg‐laying, opportunities for parasitism become limited (Abernathy and Langmore 2017). In such cases, behaviors that increase the number of suitable host nests would be advantageous to the brood parasite (Hauber 2000, 2014; Soler et al. 2017; Zhang et al. 2024). As early as 1940, Chance proposed a possible behavioral strategy for the common cuckoo ( *Cuculus canorus* ), suggesting that it could potentially destroy unparasitized host nests, prompting the host to re‐nest, thus increasing the chance for successful parasitism (Chance 1940). Arcese et al. (1992) proposed the cowbird predation hypothesis, a hypothesis that shared the same logic as that proposed by Chance. They suggest that brood parasites might predate or destroy non‐parasitized host nests to force the hosts to re‐nest, which may increase the chances of future parasitism (Arcese et al. 1996). As this behavior manipulates the reproductive cycles of hosts, it has been termed a “farming” strategy.

“Farming behavior” can create new opportunities for parasitism for brood parasites (Hauber 2000, 2014; Soler et al. 2017), and thus should be widespread among brood parasites. However, such behavior has only been observed in a few brood‐parasitic systems, such as the brown‐headed cowbird ( *Molothrus ater* ; Arcese et al. 1996; Hoover and Robinson 2007; Swan et al. 2015), bronzed cowbird ( *Molothrus aeneus* ; Peer and Sealy 1999), common cuckoo (Chance 1940; Kinoshita and Kato 1995; Kim and Yamagishi 1999; Zhong et al. 2019; Zhang et al. 2024), Himalayan Cuckoo ( *Cuculus saturatus* ; Su et al. 2017), Lesser Cuckoo ( *Cuculus poliocephalus* ; Tojo and Nakamura 2021), and Asian Koel ( *Eudynamys scolopaceus* ; Zhou and Liang 2023). Among these brood parasites, farming behavior has been more frequently documented in the brown‐headed cowbird and common cuckoo, whereas reports for other brood parasitic birds remain scarce. China hosts one of the richest diversities of parasitic bird species globally, including at least 17 species of parasitic cuckoos (Zheng 2023). However, only four cases of farming behavior by brood parasites have been reported within this country, involving three cuckoo species. No documentation of farming behavior exists for other brood parasitic birds. Detailed observation and reporting of farming behavior across different brood parasite species and systems will help deepen our understanding of coevolution between brood parasites and hosts, as well as the evolutionary significance of farming behavior. Aspects that must be addressed include whether this behavior is limited to a handful of species or is common across brood parasites, and whether it is region‐specific or globally distributed. To address these questions, in this study, we provide a detailed account of farming behavior by the Large Hawk‐cuckoo (*Hierococcyx sparverioides*) toward both the Black‐streaked Scimitar Babbler ( *Pomatorhinus erythrocnemis* ), a known host, and Silver‐eared Mesia ( *Leiothrix argentauris* ), a potential host, in southwestern China.

## 2 Methods

### 2.1 Study Area and Study Species

The study site is located in Caihu Village (23°56′‐24°29′N, 100°22′‐101°15′E), Jingdong County, in the south‐central part of Yunnan Province. Within Jingdong County, there are two national‐level nature reserves: Ailaoshan and Wuliangshan. This area is known for its rich biodiversity and abundance of endemic species, making it one of the most biologically diverse areas in Yunnan Province (Luo et al. 2011).

The Large Hawk‐cuckoo belongs to the order Cuculiformes, family Cuculidae, and is a brood‐parasitic species. Ten host species have been recorded in China, namely, the Streak‐breasted Scimitar Babbler ( *Pomatorhinus ruficollis* ), White‐browed Laughingthrush (*Pterorhinus sannio*), Chinese Babax (*Pterorhinus* lanceolatus), Elliot's Laughingthrush ( *Trochalopteron elliotii* ), White‐bellied Redstart (*Luscinia phoenicuroides*), Chinese Hwamei ( *Garrulax canorus* ), Oriental Magpie (*Pica serica*), Masked Laughingthrush (*Pterorhinus perspicillatus*), Black‐streaked Scimitar Babbler, and Mustached Laughingthrush (*Ianthocincla cineracea*) (Liu et al. 2025). During the breeding season, it inhabits broadleaved woodlands, deciduous, especially oak, evergreen, and thickets. Its main food source is insects, such as caterpillars, including hairy species, beetles, Hemiptera, ants, grasshoppers, crickets, cockroaches; spiders; birds' eggs; berries (Erritzøe et al. 2012).

The Silver‐eared Mesia is a member of the order Passeriformes, family Leiothrichidae. In China, it is primarily distributed in southern Guizhou and western, southern, and eastern Yunnan, as well as in southern Guangxi and southeastern Tibet (Zheng 2023). The breeding season spans from May to July, and the nests are usually cup‐shaped, with 3–5 eggs (Zhao 2001), often placed in understory shrubs. To date, there are no records of the Silver‐eared Mesia being parasitized by the Large Hawk‐cuckoo in China (Liu et al. 2025). The Silver‐eared Mesia builds an open cup‐shaped nest and forages on insects, including those in the orders Lepidoptera, Odonata, and Orthoptera (Liu et al. 2024). These ecological traits make it a potential and suitable host for parasitic cuckoos, for example, the Large Hawk‐Cuckoo.

The Black‐streaked Scimitar Babbler belongs to the family Timaliidae (order Passeriformes) (Zheng 2023). The breeding season spans from May to July. It nests on earthen banks, in shrub thickets, grassy areas, and bamboo forests, constructing spherical‐shaped nests with side entrances (Liu 2024). In China, this species is known to be parasitized by the Large Hawk‐cuckoo. The eggs of both species are highly similar in appearance, typically pure white in color (Liu et al. 2025; Liu and Yang 2025).

### 2.2 Field Observations and Video Recordings

**FIGURE 1:**
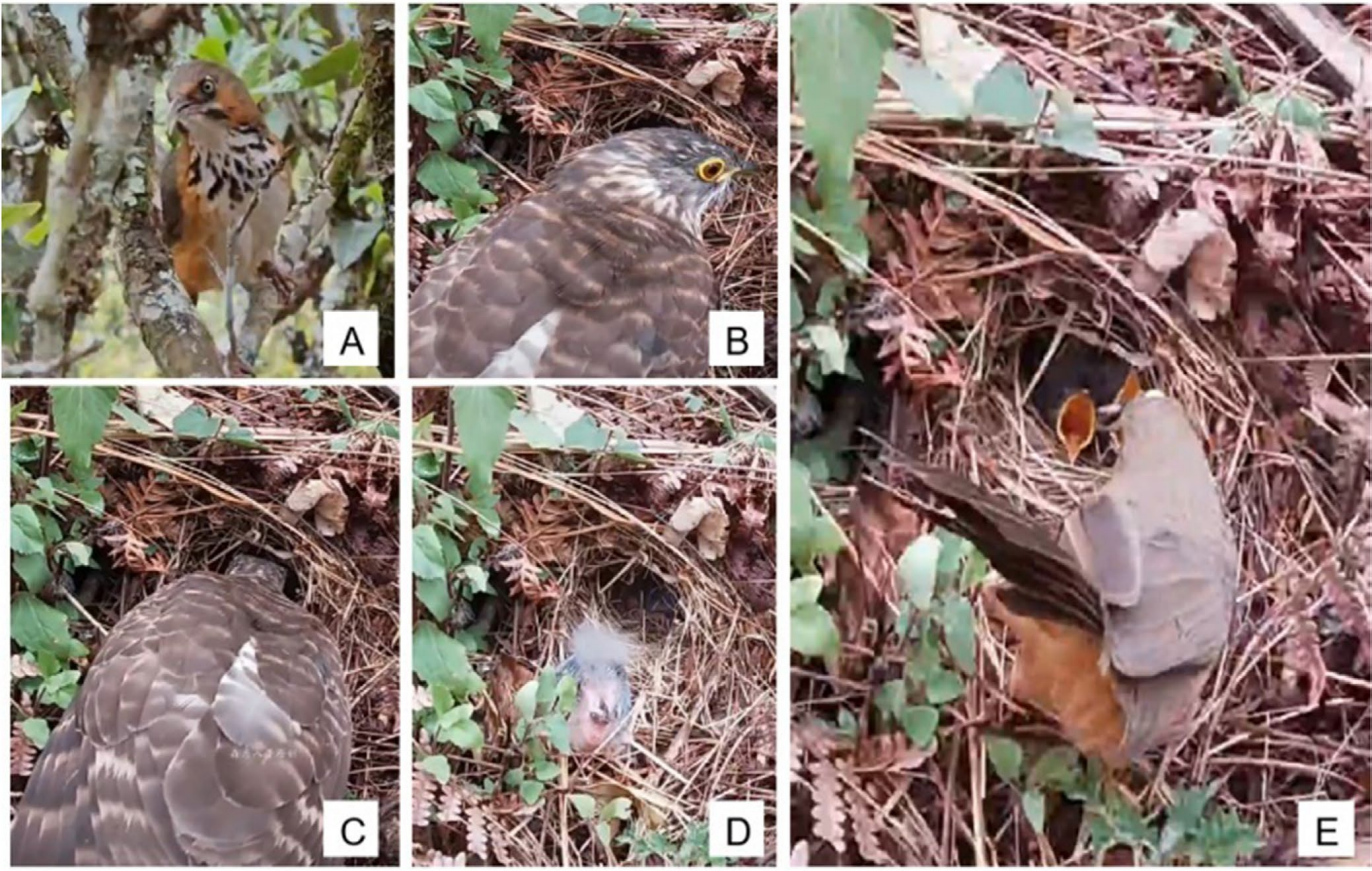
Farming behavior of the Large Hawk‐cuckoo toward the Black‐streaked Scimitar Babbler. (A) Black‐streaked Scimitar Babbler. (B) Large Hawk‐cuckoo at the nest entrance. (C) Large Hawk‐cuckoo removing the chick. (D) Chick after removal. (E) Parent babbler returns and feeds the chick.

From mid‐April to mid‐July 2024, we conducted fieldwork in the study area, systematically searching for bird nests. On April 1, 2024, we discovered a spherical nest with a side‐entrance on the ground in a tea plantation. The nest contained a single pure white egg. Based on our field experience, we identified it as a nest of the Black‐streaked Scimitar Babbler. On April 4, when observed again, three eggs were present in the nest. On the same day, we videotaped the nest using a GoPro HERO 9 action camera (GoPro, San Mateo, CA, USA) combined with a 10,000‐mAh external power supply (TP‐D094, Pisen, Guangdong Pinsheng Electronics Co. Ltd., China) for further species identification. To study egg incubation and brood rearing behaviors, we videotaped the nest for 4 h on each subsequent day during 8:00–18:00. The equipment was camouflaged with vegetation, placed approximately 1 m from the nest, and mounted on a tea tree to minimize disturbance. On April 22, at 11:20:16, a Large Hawk‐cuckoo arrived at the nest, where there were three chicks. After observing at the nest edge for 14 s, it picked up a 7‐day‐old Black‐streaked Scimitar Babbler chick at 11:20:40 and flew away with it. At 11:20:42, the chick crawled back into the nest. While the Large Hawk‐cuckoo was near the nest, the Black‐streaked Scimitar Babbler parent stayed on guard in a tree near the nest. When the chick climbed back to the nest, the Black‐streaked Scimitar Babbler parent returned to the nest and continued to feed the chick (Figure 1). The nestling eventually fledged successfully.

**FIGURE 2:**
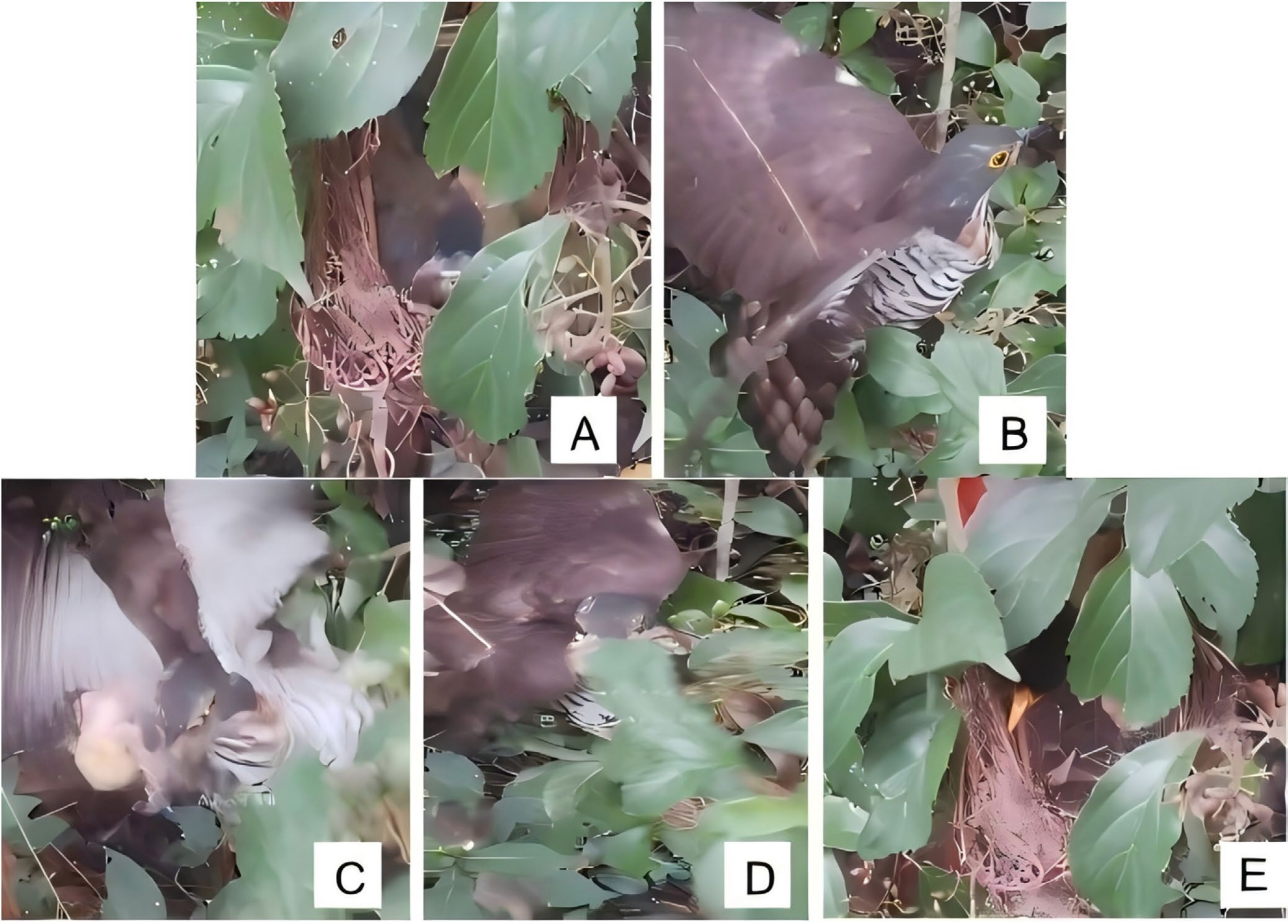
Farming behavior of the Large Hawk‐cuckoo toward the Silver‐eared Mesia. (A) Silver‐eared Mesia chick. (B) Large Hawk‐cuckoo. (C) Cuckoo ejecting one chick. (D) Cuckoo ejecting the remaining two chicks. (E) Silver‐eared Acacia (♀) returning to the nest.

On May 1, 2024, we found a Silver‐eared Mesia nest in a small tree in the forest. The nest was open‐cup shaped, with a regular top opening. The nest was approximately 1.5 m above the ground, and mainly comprised dry grass, stems, and fibrous roots. It contained one egg with a white base color and sparse reddish‐brown speckles at the blunt end. When we revisited the nest on May 5, three eggs were present. We also videotaped the nest on the same day, with the same methodology as specified above. On May 15, all three eggs successfully hatched. On May 17 at 12:45:12, a Large Hawk‐cuckoo arrived at the Silver‐eared Mesia nest. Within 10 s, it threw all three approximately 12‐day‐old chicks out of the nest and flew away without consuming the fallen chicks (Figure 2). Fifteen minutes later, the female Silver‐eared Mesia returned, found the nest destroyed, and abandoned it (Figure 2).

## 3 Discussion

In this study, we present the first documented cases of the Large Hawk‐cuckoo removing nestlings from the nests of both the Black‐streaked Scimitar Babbler and Silver‐eared Mesia. In the case of the ground‐nesting Black‐streaked Scimitar Babbler, the chick removed by the Large Hawk‐cuckoo was not killed. As the chick was relatively large and the cuckoo did not further harm it, the chick could crawl back into the nest, where it continued to be fed by the parent until it successfully fledged. In contrast, the nestlings of the Silver‐eared Mesia, which nests above ground, likely died after being ejected. When we subsequently checked the nest, the ejected Mesia chicks were no longer visible, and we suspect they were captured by predators.

There are at least three non‐mutually exclusive hypotheses to explain the destruction or predation of host eggs or chicks by nest parasitic birds. According to the nutritional farming hypothesis (Wyllie 1981), brood parasites, such as certain nest predators, consume the eggs or chicks of other birds to gain nutritional benefits (Davies and Brooke 1988; Manzer and Hannon 2005; Su et al. 2017). However, our study does not support this hypothesis. Similar to other documented cases, the Large Hawk‐cuckoo did not consume the host's eggs or chicks but only destroyed or removed them (Chen et al. 2009; Šulc et al. 2020; Tojo and Nakamura 2021; Zhou and Liang 2023). The second is the mafia (or mafia retaliatory) hypothesis. It suggests that parasitic birds destroy or predate host nests as a form of punitive retaliation against hosts that reject their parasitic eggs. The brood parasite monitors previously parasitized nests, and if it finds its eggs or chicks missing, it retaliates by damaging or predating the nest (Zahavi 1979; Soler et al. 1995). Although the mafia hypothesis has received considerable theoretical and experimental support (Soler et al. 1995; Hoover and Robinson 2007; Hauber 2009; Abou Chakra et al. 2016), our study does not support the hypothesis. Given that neither the Silver‐eared Mesia nor the Black‐streaked Scimitar Babbler nests contained Large Hawk‐Cuckoo eggs or chicks during our monitoring, we conclude that brood parasitism did not take place. Therefore, no retaliatory response would have been triggered by egg rejection (Hoover and Robinson 2007). The third is the farming hypothesis, wherein brood parasites destroy host nests that are no longer suitable for parasitism (e.g., nests in late incubation or chick‐rearing stages) by removing eggs and/or chicks. This forces the host to re‐nest, thus creating new opportunities for parasitism. This strategy has been confirmed in both the Brown‐headed Cowbird and Common Cuckoo (Hoover and Robinson 2007; Swan et al. 2015; Zhang et al. 2024). For example, Zhang et al. (2024) provided video evidence of a Common Cuckoo removing nestlings from a Daurian Redstart ( *Phoenicurus auroreus* ) nest, which led to rebuilding by the host. The new nest was subsequently parasitized, offering strong evidence of farming behavior (Zhang et al. 2024). Our findings support the farming hypothesis. The Large Hawk‐cuckoo damaged the nests of Silver‐eared Mesia and displaced a Black‐streaked Scimitar Babbler nestling during their chick‐rearing stages, likely because it had missed the optimal parasitism window. By destroying nests that were no longer suitable for parasitism, the cuckoo may have induced re‐nesting, thus creating opportunities for future parasitism (Swan et al. 2015).

This is the first recorded case of unsuccessful and successful farming behaviors by the Large Hawk‐cuckoo toward ground‐ (Black‐streaked Scimitar Babbler) and tree‐nesting species (Silver‐eared Mesia), respectively. This outcome may be attributed to nest placement, as tree‐nesting species often suffer complete breeding failure if chicks fall from the nest before fledging. Although we have not yet observed direct cases of adult Large Hawk‐cuckoos killing host nestlings, such events may be more frequent than realized. In tree‐nesting species, ejected chicks quickly disappear after falling to the ground, making it difficult to distinguish between parasite‐driven and natural predation. Host eggs or chicks removed by brood parasites are rarely recovered. Relevant future research can be conducted in the following areas: First, research can be conducted across broader geographic regions and varying ecological contexts to evaluate the prevalence and adaptive variability of farming behavior in brood parasites. Second, nest‐monitoring technologies should be deployed to capture comprehensive, high‐resolution data to further document and understand parasite‐driven farming behavior in natural settings.

## Author Contributions

**Feiling Pang:** data curation (equal), formal analysis (equal), investigation (equal), writing – original draft (equal). **Keyan Zhang:** formal analysis (equal), investigation (equal), writing – original draft (equal). **Ziyu Yuan:** data curation (equal), formal analysis (equal), investigation (equal). **Jianping Liu:** funding acquisition (equal), project administration (equal), writing – review and editing (equal).

## Funding

J.L. was supported by the Ningxia Natural Science Foundation (2025AAC030051), the National Natural Science Foundation of China (No. 32160242), the 2023 Ningxia Hui Autonomous Region Youth Science and Technology Support Talent Training Project and the third tranche of the 2025 central government subsidy project allocated to the Liupanshan National Nature Reserve.

## Ethics Statement

The experiments comply with the current laws of China, where they were performed. Experimental procedures were in agreement with the Animal Research Ethics Committee of Hainan Provincial Education Centre for Ecology and Environment, Hainan Normal University (No. HNECEE‐2014‐005).

## Conflicts of Interest

The authors declare no conflicts of interest.

## Data Availability

No data used for this study. Videos are provided as (Videos S1 and S2) and can be found at https://doi.org/10.6084/m9.figshare.30461699.v1. On April 22, 2024, a Large Hawk‐cuckoo arrived at the nest of Black‐streaked Scimitar Babbler. After observing at the nest edge for 14 s, it picked up a 7‐day‐old Black‐streaked Scimitar Babbler chick at 11:20:40 and flew away with it. Video content can be viewed at https://onlinelibrary.wiley.com/doi/10.1002/ece3.72955. On May 17, 2024, a Large Hawk‐cuckoo arrived at the Silver‐eared Mesia nest. Within 10 s, it threw all three approximately 12‐day‐old chicks out of the nest and flew away without consuming the fallen chicks. Video content can be viewed at https://onlinelibrary.wiley.com/doi/10.1002/ece3.72955.
